# Challenges of Salinity Intrusion and Drought Stress on Olive Tree Cultivation on Mljet Island

**DOI:** 10.3390/plants13182549

**Published:** 2024-09-11

**Authors:** Josip Tadić, Gvozden Dumičić, Maja Veršić Bratinčević, Sandra Vitko, Sandra Radić Brkanac

**Affiliations:** 1 Institute for Adriatic Crops and Karst Reclamation, 21000 Split, Croatia; josip.tadic@krs.hr (J.T.); gvozden.dumicic@krs.hr (G.D.); maja.versic.bratincevic@krs.hr (M.V.B.); 2 Centre of Excellence for Biodiversity and Molecular Plant Breeding (CoE CroPBioDiv), 10000 Zagreb, Croatia; 3 Department of Biology, Faculty of Science, University of Zagreb, 10000 Zagreb, Croatia; sandra.vitko@biol.pmf.hr

**Keywords:** *Olea europaea*, abiotic stress, ion content, biochemical changes

## Abstract

Understanding genotype-specific responses to environmental stressors is vital for developing resilience strategies that ensure sustainable olive cultivation and productivity. In this work, cultivar ‘Oblica’ and several olive genotypes from the island of Mljet (Croatia) were exposed to short-term (21 days) salinity and drought treatments. In contrast to other olive genotypes, genotype M29 as well as cultivar ‘Oblica’ managed to maintain growth and chlorophyll *a* levels under salinity stress to the same level as the control. Drought, however, significantly reduced the growth parameters in all olive trees. Cultivar ‘Oblica’ accumulated the greatest amount of Na^+^ ions in the leaves compared to olive genotypes from the island of Mljet, demonstrating superior resistance by translocating Na^+^ to leaf vacuoles. The observed reduction in K^+^ content in the roots of olive trees under all treatments suggests a generalized stress response. On the other hand, effective Ca^2+^ uptake has been identified as a crucial energy-saving strategy that olive trees use to cope with brief periods of salinity and drought. The proline content and activities of superoxide dismutase (SOD) and guaiacol peroxidase (GPOX) varied among the olive trees, highlighting the importance of antioxidative capacities and stress adaptation mechanisms. According to the obtained results, stress-resistant olive genotypes like ‘Oblica’ and M29 show potential for breeding resilient varieties.

## 1. Introduction

The island of Mljet, often called the ‘green island’ due to its lush vegetation, lies within the Mediterranean climatic region. This area is characterized by warm, dry summers and mild winters with significant rainfall [1]. Despite the island’s lack of impermeable substrates, which results in the absence of surface water flow, the northwest region is home to the Veliko and Malo lakes—submerged karst valleys filled with seawater [2]. Mljet features freshwater springs and brackish water pools, known locally as ‘blatine’. These pools support eels, indicating a direct connection to the sea through intricate underground fissures and channels [3]. Despite high annual precipitation, coastal areas often experience saline soils due to rising sea levels, seawater intrusion, and salinization from sea spray carried by the wind [4]. This rise has led to saline groundwater intrusion inland, resulting in contact with non-saline surface water or, in extreme cases, penetration into the soil profile within the plant root zone [5,6]. The island serves as a model for studying the effects of increased soil salinity, given its vulnerability to salinity intrusion from the sea. It is one of eight strategically selected locations along the Adriatic coast, chosen for their unique vulnerabilities and to provide comprehensive insights into regional patterns of saline intrusion. Poor quality irrigation water, with salinity greater than 2 dS/m, further exacerbates soil salinization in agricultural production [7,8]. Natural habitats and agricultural lands are facing an increasing problem due to water scarcity caused by extended periods of drought and lower precipitation levels. This leads to soil erosion and the spread of deserts in the Mediterranean region, especially in southern Europe and northern Africa [9,10]. State-of-the-art models have confirmed a significant increase in drought frequency in the Mediterranean basin, partly driven by rising greenhouse gas emissions [10,11]. The expansion to previously unfavorable conditions, such as the progressive movement towards the north, has become a viable option for olive cultivation in the changing climate of the Mediterranean [12,13].

Exposure to severe water deficit, often caused by excessive salt concentrations, produces an osmotic imbalance in plants, significantly reducing total carbon assimilation and biomass production [14]. Besides osmotic stress, olive trees experience oxidative stress due to increased salinity and drought, with the intensity depending on salinity levels, Na^+^ and Cl^−^ concentrations, and the duration of drought [15,16]. In response to these stresses, olive trees adjust osmotically by increasing the uptake of low energy-cost inorganic ions, primarily K^+^. This adaptation is seen across both resistant and sensitive cultivars [17], accompanied by the accumulation of osmoprotectants [18]. Indicators of osmotic stress in young olive leaves include the concentration of K^+^, proline, other compatible solutes, and inorganic ions [17,19]. Genotypic variation in response to osmotic stress is observed across plant species, including olive trees [18,20]. To find potential candidates for developing olive cultivars more resistant to challenging environmental conditions (like extended drought events and increase in soil salinity over summer), we have conducted a comprehensive short-term, high-stress intensity experiment examining over 26 olive genotypes, with emphasis on Croatian native cultivated and wild genotypes from islands and peninsulas on the Adriatic Sea (Croatia) [21,22,23,24]. Part of the data obtained by the research showed that not all wild olive genotypes inherently ensure superior plant traits under unfavorable environmental conditions [21,23]. Here, we highlight olive genotypes from Mljet island, given the distinct environmental challenges they face. The primary concern on the island is the rising sea levels resulting in saline groundwater intrusion, posing direct risks to soil quality and agricultural productivity. Understanding water stress is another critical objective, investigating the relationship between drought events, reduced rainfall, and soil degradation on the island. Mitigating these effects is vital for maintaining the island’s ecological balance and sustaining its agricultural output. From the island, 30 samples were collected to represent the olive genotypes present there. Our focus was on genotypes that had successfully rooted from at least 15 cuttings per genotype. Through genetic relationship analysis using SSR markers, we discovered that many genotypes initially presumed to be wild olives (*Olea europaea* subsp. *europaea* var. *sylvestris*) are, in fact, closely related to cultivated varieties [23,24]. Including this information underscores the significance of Mljet’s unique location and its genetic diversity of olives, making it an ideal site for studying the mechanisms of salt and drought tolerance [25]. The study aimed to investigate olive genotypes from Mljet island in their natural habitat to assess their potential for enhanced salt and drought stress resistance compared to the well-known cultivated olive genotype ‘Oblica’. To achieve this objective, various parameters were measured, including growth (shoot length, leaf surface area, shoot dry mass), photosynthetic performance (chlorophyll *a*), salt ion accumulation, mineral composition (K^+^, Ca^2+^, Mg^2+^), stress indicators (malondialdehyde, proline) and antioxidative defense mechanisms (SOD, GPOX).

## 2. Results

### 2.1. Influence of Salinity and Drought on Growth and Photosynthetic Pigments

**Figure 1:**
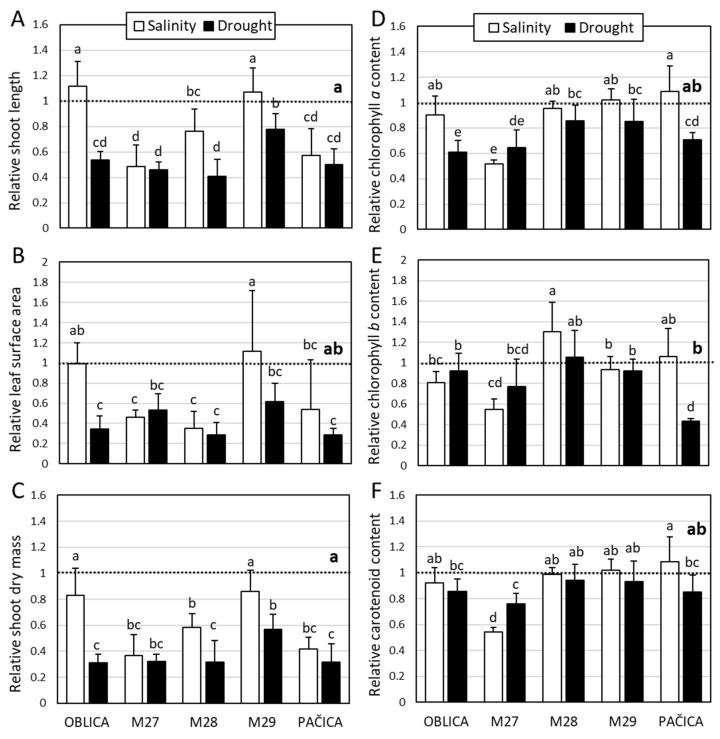
Relative shoot length (**A**), leaf surface area (**B**), shoot dry mass (**C**), chlorophyll *a* (**D**), chlorophyll *b* (**E**), and carotenoids (**F**) of olive genotypes under short-term salinity and drought. Controls of olive genotypes are set to the value 1 (dashed line). Presented data are means of three replicates ± SD. Bars and dashed line (bold) labeled with different letters are significantly different at *p* ≤ 0.05. Raw data (absolute values) are presented in Supplementary Tables S1 and S2.

Three olive genotypes and a lesser known cv. ‘Pačica’ from island Mljet were selected for physiological and biochemical characterization under drought or salinity stress. Selected genotypes were analyzed and compared with reference cv. ‘Oblica’. Aside from chlorophyll *b* and carotenoids, in this experiment drought had a more significant effect on growth and chlorophyll *a* levels in most olive genotypes compared to salinity (Figure 1). Growth parameters and chlorophyll *a* content of almost all olive genotypes significantly decreased under drought treatment. Drought did not significantly affect the leaf surface area of M27 and M29 genotypes or the chlorophyll *a* content of M28 and M29 genotypes, compared to control (Figure 1B,D). Chlorophyll *b* and carotenoids significantly declined under drought stress only in the cv. ‘Pačica’ and M27 genotypes, respectively, compared to the control (Figure 1E,F). Shoot length and shoot dry mass of salt-treated cv. ‘Oblica’ and genotype M29 were not different from the control. Other genotypes, including cv. ‘Pačica’, demonstrated a marked decrease in those growth parameters when faced with salinity stress (Figure 1A,C). In contrast to other olive genotypes, the leaf surface areas of the salt-treated cv. ‘Oblica’, genotype M29 and cv. ‘Pačica’ were similar to control (Figure 1B). The salinity treatment reduced chlorophyll *a* content only in the M27 genotype (Figure 1D). Of all the genotypes, only the salt-stressed M27 genotype had significantly declined chlorophyll *b* and carotenoids compared to the control.

### 2.2. Influence of Salinity and Drought on Leaf and Root Levels of Na^+^ and Cl^−^

**Figure 2:**
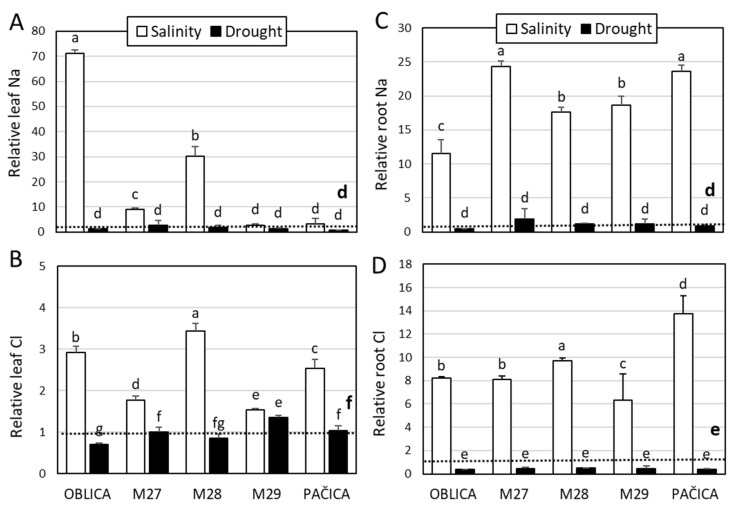
Relative content of leaf and root Na^+^ (**A**,**C**), and Cl^−^ (**B**,**D**) for olive genotypes under short-term salinity and drought. Controls of olive genotypes are set to the value 1 (dashed line). Presented data are means of three replicates ± SD. Bars and dashed line (bold) labeled with different letters are significantly different at *p* ≤ 0.05. Raw data (absolute values) are presented in Supplementary Table S3.

A significant change in Na^+^ and Cl^−^ was noted in both shoot leaves and roots (Figure 2). Regarding the accumulation of leaf Na^+^, the highest content of the ion was noted in cv. ‘Oblica’ followed by genotypes M28 and M27 (Figure 2A). Leaf Na^+^ levels of other salt-treated olive genotypes were comparable to control. Salinity treatment significantly increased leaf Cl^−^ content levels in all olive genotypes, especially in genotype M28, cv. ‘Oblica’ and ‘Pačica’ (Figure 2B). Leaf Na^+^ and Cl^−^ values were not affected by drought. A significant decrease in leaf Cl^−^ content compared to the control was noted only in cv. ‘Oblica’ (Figure 2B). All olive varieties, especially genotype M27 and cv. ‘Pačica’, experienced a considerable rise in root Na^+^ levels due to salinity treatment (Figure 2C). Roots of all genotypes, especially cv. ‘Pačica’, showed significantly increased Cl^−^ values under salinity (Figure 2D). The values of salt ions in roots were similar to those in the control treatment.

### 2.3. Influence of Salinity and Drought on Leaf and Root Levels of K^+^, Mg^2+^ and Ca^2+^

Irrespective of the treatment, leaf Mg^2+^ content was more negatively affected than the root values of those ions. In contrast, the leaf K^+^ content was less affected than the root content in most olive genotypes regardless of the applied treatment. Leaf Mg^2+^ content in cv. ‘Oblica’ significantly decreased under both treatments and that in M28 and M29 genotypes only under salinity treatment (Table 1). Only cv. ‘Oblica’ had significantly reduced Ca^2+^ content under both treatments. Significantly lower values for K^+^ content were noted in cv. ‘Oblica’ under salinity treatment and in cv. ‘Pačica’ under drought treatment. Salinity increased the values of K^+^ leakage in olive genotypes in comparison to the control, though the rise was only significant in cv. ‘Oblica’ (Table 1).

Mg^2+^ content in the roots of olive genotypes showed either increased values or values similar to the control treatment (Table 2). A similar trend was noted for Ca^2+^ content apart from genotype M27 and cv. ‘Pačica’, which showed significantly decreased values for that macroelement under salinity treatment. All genotypes experienced a decline in the root K^+^ values (Table 2) regardless of the treatment.

### 2.4. Influence of Salinity and Drought on Biochemical Indicators

**Figure 3:**
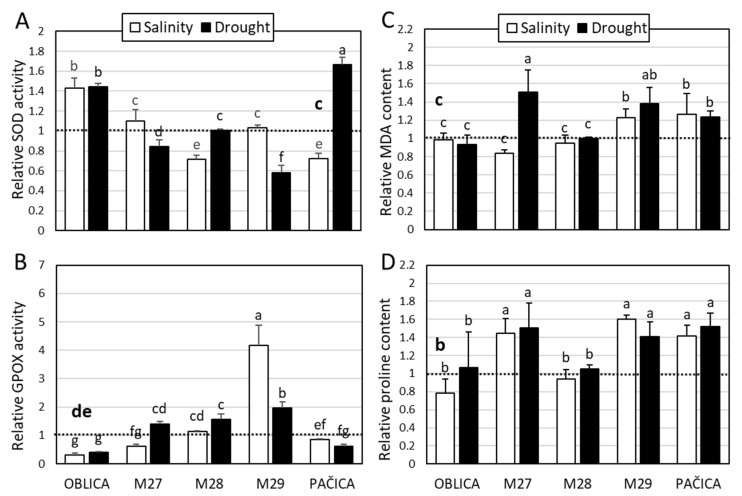
Relative activity of SOD (**A**), and GPOX (**B**), relative MDA (**C**), and proline (**D**) content in olive leaves under short-term salinity and drought. Controls of olive genotypes are set to the value 1 (dashed line). Presented data are means of three replicates ± SD. Bars and dashed line (bold) labeled with different letters are significantly different at *p* ≤ 0.05. Raw data (absolute values) are presented in Supplementary Table S6.

The activity of SOD was stimulated in cv. ‘Oblica’ under both treatments and in cv. ‘Pačica’ under drought treatment (Figure 3A). The activity of that antioxidative enzyme was either similar to control or inhibited in other genotypes under salinity or drought (Figure 3A). Induction of GPOX was recorded in genotype M29 under both treatments and in genotype M28 under drought treatment (Figure 3B). Salinity caused a significant inhibition of GPOX activity in the reference cv. ‘Oblica’ and genotype M27. A decline in activity of that enzyme was detected under drought stress in cv. ‘Pačica’ (Figure 3B). An indicator of lipid peroxidation, MDA content, exhibited greater changes under drought than salinity treatment. In contrast to cv. ‘Oblica’ and genotype M28, cv. ‘Pačica’ and genotype M29 displayed a marked buildup of MDA in response to both treatments (Figure 3C). Both stress factors increased the proline content in cv. ‘Pačica’ and genotypes M27 and M29 (Figure 3D).

### 2.5. Evaluation of the Tolerance of Olive Genotypes to Salinity and Drought Using PCA

**Figure 4:**
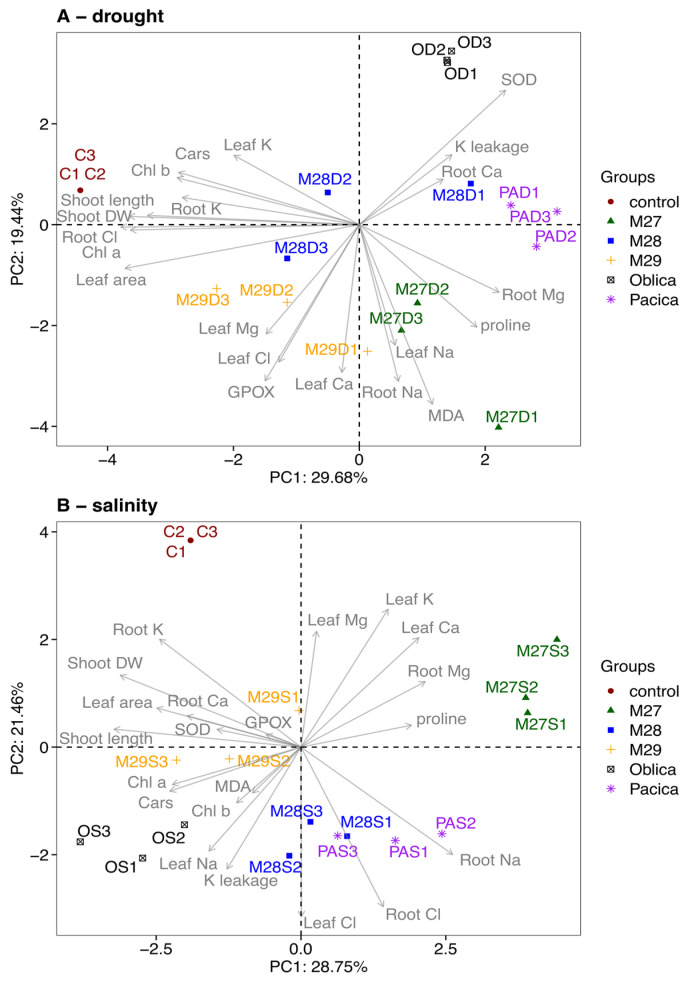
Principal component analysis (PCA) biplot of olive response to drought (**A**) and salinity (**B**). Biplots show the relationships between different olive genotypes (M27, M28, M29, Oblica, Pačica) and the measured morphological (shoot length and dry mass, leaf surface area), physiological (chlorophyll *a*, chlorophyll *b* and total carotenoids content) and biochemical (SOD and GPOX activity, proline and MDA content, K^+^ leakage, Na^+^, Cl^−^, K^+^, Mg^2+^ and Ca^2+^ ion content in leaves and roots) variables under each stress condition. Controls of olive genotypes are normalized to the value 1.

To better understand the complex relationships between genetic background and olive response to salinity and drought stress, and to identify the variables contributing to the differences observed between these treatments, we used principal component analysis (PCA). Preliminary PCA showed that the primary factor influencing the observed variations in the data was the treatment conditions rather than the differences between the genetic backgrounds of the olive genotypes (Supplementary Materials, Figure S1). In other words, drought stress had a stronger effect than salinity and was characterized by a greater reduction in olive tree developmental metrics compared to salinity, including shoot length and dry mass, leaf area, and pigment content. In addition, the response to drought stress was characterized by a greater decrease in K^+^ leakage and ion content in leaves (Na^+^, Cl^−^) and roots (Na^+^, Cl^−^, K^+^). At the same time, the content of Mg^2+^ and Ca^2+^ increased in both roots and leaves. To further elucidate genotype-specific responses to drought and salinity, we generated separate PCA biplots for each stress condition (Figure 4A,B). A biplot generated for drought response analysis confirmed that drought-stressed plants were significantly different from control plants. All drought-treated genotypes showed reduced shoot length and dry mass, leaf area, pigment content, and K^+^ content in leaves and roots (Figure 4A). Of the genotypes tested, M29 was the most resistant to drought, with relatively less growth reduction compared to the other genotypes and with increased GPOX activity and Cl^−^ content in leaves. In contrast, cv. ‘Oblica’ was the most sensitive to drought. This genotype was characterized by lower GPOX activity and lower Cl^−^, Mg^2+^ and Ca^2+^ content in leaves, but higher SOD activity, higher K^+^ leakage and higher Ca^2+^ content in roots. The response to salinity differed somewhat, with the tcv. ‘Oblica’ and M29 genotype showing the highest tolerance (Figure 4B). Looking at developmental indicators (shoot length, dry mass, leaf area, and pigment content), these two genotypes did not differ significantly from the control plants. However, they showed different responses to salinity, as documented by differences in enzyme activities and ion content. Cv. ‘Oblica’ was characterized by higher SOD activity, K^+^ leakage, Na^+^ content in leaves, and Cl^−^ content in leaves and roots. In contrast, M29 showed higher proline level, GPOX activity and Na^+^ content in roots. The highest sensitivity to salinity was shown by genotype M27, followed by a lower proportion of Cl^−^ and Ca^2+^ ions in the leaves, and a higher content of proline and Na^+^ and Mg^2+^ in the roots.

## 3. Discussion

### 3.1. Influence of Salinity and Drought on Growth and Photosynthetic Pigments

Many evergreen sclerophyllous plants, such as olives, evolved in environments characterized by drought stress and nutrient-poor soils. These plants inherently exhibit slower rates of CO_2_ assimilation and biomass production, making them less affected by the osmotic factors of increased salinity [26].

In this study, while the ‘Oblica’ cultivar was expected to show reduced morphometric measurements [19], most values did not significantly differ from the control treatment. Consistent findings were reported by Bashir et al. [27], documenting reduced shoot growth at even lower NaCl concentrations (50 mM) in resistant and sensitive cultivars, indicating prevalent osmotic stress with minimal genotype variation. Perica et al. [19] observed that increased salinity had a linear effect on growth parameters in olive cultivars (including cv. ‘Oblica’), suggesting an inversely proportional relationship between salinity resistance and plant vigor [28]. Using mannitol to induce drought did not result in positive morphometric and biochemical changes, indicating that mannitol primarily acted osmotically, accumulating outside root cells and affecting root dry weight.

Chlorophyll *a* is crucial for photosynthesis, and its reduction under drought stress indicates impaired photosynthetic efficiency, leading to decreased carbohydrate production and energy availability for growth [29]. Drought induced by mannitol had a stronger effect on chlorophyll *a* than salinity across most olive genotypes. The genotype M27 exposed to either stress showed the most noticeable decrease in chlorophyll *a* content among olive trees from Mljet. Abdallah et al. [30] also reported a statistically significant reduction in chlorophylls content in the cv. ‘Chétoui’ under increased salinity and drought. In our study, the chlorophyll *a* content of drought-treated cv. ‘Pačica’ was also reduced, while the values of that photosynthetic pigment did not change under the influence of salinity. That result is consistent with Perica et al. [19], who reported high relative chlorophyll content in cv. ‘Pačica’ exposed to salinity. Similarly, Ayaz et al. [31] found that drought stress using PEG 6000 had stronger effects on chlorophylls in the cv. ‘Ayvalik’ than salinity stress. These findings highlight the critical role of chlorophyll *a* in photosynthesis and its vulnerability to drought stress, which more severely impairs photosynthetic efficiency and carbohydrate production than salinity [18]. The greater impact of mannitol-induced drought on chlorophyll *a* content of olive genotypes suggests that water scarcity poses a more serious threat to photosynthetic activity than salinity [32,33].

### 3.2. Influence of Salinity and Drought on Leaf and Root Levels of Na^+^ and Cl^−^

The second phase of reduced growth in olive trees is linked to the advanced aging of leaves caused by excessive accumulation of Na^+^ and Cl^−^ in all transpiring leaves. This accumulation leads to premature aging, diminishing the plant’s photosynthetic capacity to a level insufficient for continued growth [15]. This phenomenon is known as the ‘low sodium strategy’ [16,34]. Diminished water transport from the root, along with increased NaCl concentration and moderate growth dynamics in *Olea europaea* L., may enhance its ability to protect shoots sensitive to high levels of toxic salt ions. The ‘Oblica’ cultivar utilizes a similar resistance mechanism [20] as the leaf Na^+^ levels were several folds higher compared to other genotypes. Interestingly, the salinity did not influence the growth of ‘Oblica’ cultivar. Such results are probably associated with relatively efficient vacuolar Na^+^ accumulation, which helped maintain the cells’ osmotic pressure. In contrast to drought, harmful Na^+^ and Cl^−^ ions accumulated to significantly high levels in olive leaves and roots exposed to salinity. Cl^−^ likely contributes to osmotic adjustment, although to a lesser extent than Na^+^ [20]. This could explain why more sensitive cultivars, such as ‘Leccino’, quickly respond to stress by reducing shoot growth intensity, thereby preventing the transport of harmful Na^+^ ions to their sensitive leaves [17,19,20]. In the study by Tadić et al. [22], wild olive genotypes accumulated most of the Na^+^ and Cl^−^ ions in their root systems to protect shoot growth and young leaves. However, unlike the cultivar ‘Oblica’, their shoot and leaf surface development was halted, indicating an early perception of stress. Traits such as well-developed root systems are essential in resistance to salinity and drought [35]. Ziogas et al. [36] stated that drought and salinity had distinct effects on the ion content in the root and leaf tissues of citrus trees. Their findings confirmed that drought primarily impacts water uptake and transport efficiency, whereas salinity stress necessitates complex ion regulation mechanisms to avoid toxicity. Understanding the mechanisms of ion uptake and distribution, along with genotype-specific responses, is crucial in developing comprehensive strategies for enhancing olive tree resilience to abiotic stresses.

### 3.3. Influence of Salinity and Drought on Leaf and Root Levels of K^+^, Mg^2+^ and Ca^2+^

Olive trees (*Olea europaea* L.) exhibit a variety of adaptive strategies to navigate and survive abiotic stresses such as salinity and drought, which are crucial for their growth and productivity. According to Tattini and Traversi [14], ion accumulation plays a critical role in osmotic adaptation under these stressful conditions, particularly the accumulation of K^+^ and Ca^2^^+^. Maintaining essential nutrients like Mg^2^^+^ and K^+^ in the leaves is vital for photosynthetic efficiency and sugar translocation, directly influencing crop yield and quality [37]. Interestingly, despite showing lower leaf Mg^2+^ and Ca^2+^ levels under salinity and increased K^+^ leakage, cv. ‘Oblica’ succeeded in maintaining growth similar to the control values. Such results might be explained by the relatively undisturbed levels of leaf K^+^ and probable vacuolar sequestration of salt ions (predominantly Na^+^) utilized for osmotic adjustment in leaf cells. On the other hand, most salt-treated olive trees from Mljet (apart from M28) prevented translocation of Na^+^ from roots to shoots keeping leaf Mg^2+^ and Ca^2+^ levels almost similar to control ones, which however seems to be inconsequential for the growth performance of those olive trees.

Reactive oxygen species (ROS) contribute to K^+^ efflux from plant cells, a measure of stress-induced injury in plant tissues [37]. Increased ROS generation can facilitate K^+^ leakage by the activation of protein channels under stress conditions [38]. Several studies, including Wang et al. [39], have suggested that the uptake of K^+^ from the soil is inhibited during the osmotic phase of stress, leading primarily to ion stress induced by Na^+^ and Cl^−^ ions. The variability in K^+^ uptake among olive genotypes under high salinity is attributed to differences in proton pump activity in root cells, which influences overall ion balance [40]. Chartzoulakis [16] proposed that olives manage excessive salt accumulation by preferentially accumulating toxic ions in older leaves, maintaining high K^+^ levels in younger leaves, which is crucial for osmotic regulation under high salinity. This suggests that these olive varieties can sustain leaf K^+^ content under salinity and probably utilize this ion in the cytoplasm to mitigate cellular water loss [41]. The differential effects of NaCl and mannitol on olive growth highlight the energetically favorable use of Na^+^ and Cl^−^ for osmotic adjustment, provided that these ions are sequestered in vacuoles, minimizing energy expenditure compared to the synthesis of organic solutes [42].

### 3.4. Influence of Salinity and Drought on Biochemical Indicators

Analysis of the biochemical indicators of the stressed olive genotypes demonstrates significant differences in antioxidative enzyme activities and other biochemical markers. Na^+^ begins to negatively affect enzymes at concentrations over 100 mM, while the toxic level of Cl^−^ is less defined but is likely similar to Na^+^ [43]. Drought stress induced greater activity by the SOD enzyme in the olive cv. ‘Oblica’ and ‘Pačica’ compared to salinity stress. Conversely, other genotypes did not exhibit increased SOD activity under either stress condition. Increased SOD activity serves as the initial defense against ROS, as this antioxidant enzyme facilitates the conversion of superoxide radicals into oxygen [44]. In the study by Goreta et al. [17], the cultivar ‘Leccino’ showed an initial increase in SOD enzyme activity soon after exposure to salinity, which decreased with prolonged exposure to stress. An opposite pattern in SOD activity was detected in cv. ‘Oblica’. Such results imply that cv. ‘Oblica’ can adapt more efficiently to salt stress than cv. ‘Leccino’. In long-term experiments with increased salinity, other signs of stress can appear, reducing SOD enzyme activity as hydrogen peroxide is further degraded by catalase and peroxidases [45].

GPOX activity showed a complex pattern, with significant induction in genotype M29 under both stress conditions and in genotype M28 under drought stress. Otherwise, GPOX activity was notably inhibited in most other genotypes, including cv. ‘Oblica’. The differential activity of antioxidative enzymes such as SOD and GPOX under stress conditions indicates that plants might allocate resources differently to cope with these stresses, affecting metabolism and potentially reducing photosynthesis. Although most genotypes showed increased SOD enzyme activity in both treatments, GPOX activities were reduced, but the relative content of malondialdehyde (MDA) increased depending on the genotype and treatment. In previous work by Tadić et al. [22], while SOD activity fluctuated between cultivated and wild olives, the levels of GPOX activity and MDA content remained elevated only in wild olive genotypes, regardless of the treatment. Similar to our results, treatments with increased salinity and drought increased lipid peroxidation in the ‘Chétoui’ cultivar after 21 days [30]. In a recent study by Ayaz et al. [31], statistically significantly higher MDA values were recorded for the olive cv. ‘Ayvalik’, but only in the treatment with the highest concentration of 300 mM NaCl after 15 days. By the 30th day, MDA values statistically significantly decreased compared to the control treatment, suggesting that other enzymes, such as catalase and peroxidases, may be involved in detoxifying hydrogen peroxide. In this study, salinity and drought stress contributed to a higher level of oxidative stress, highlighting the difficulty in maintaining consistent osmotic potential under varying irrigation regimes. The inhibition of critical enzymes like GPOX and the increase in stress markers such as MDA and proline reflect underlying damage and metabolic disturbances that compromise plant productivity.

Proline content, another stress marker, was generally increased in response to drought and salinity, except in cv. ‘Oblica’ and genotype M28. Besides acting as an excellent osmoprotectant, proline has roles as a metal chelator, antioxidant, and signaling molecule [46]. Interestingly, most genotypes except for genotype M28 and cv. ‘Oblica’, had increased proline concentrations. Genotypes M27, M29, and the cv. ‘Pačica’ exhibited consistently high proline levels regardless of treatment. Similarly, proline accumulation was noted in the study of Regni et al. [47]. In contrast, Ben Ahmed et al. [48] found proline levels increased in olives exposed to higher salinity. Bashir et al. [27] found increased proline levels in the resistant cultivar ‘Canino’ compared to the sensitive ‘Sirole’ in control treatments. At low concentrations (50 mM NaCl), ‘Sirole’ had higher proline levels, but at 200 mM NaCl, proline content was higher in ‘Canino’, though lower than in controls. Tadić et al. [22] also discovered that proline levels varied significantly among wild olive genotypes within the mentioned subspecies variety, regardless of the SOD, GPOX, and MDA results. These studies suggest that proline content alone may not serve as a reliable stress indicator. However, monitoring proline levels during the early stages of stress exposure is critical, as prolonged exposure can lead to statistically significant decreases in proline content [17,29,31]. Bashir et al. [27] emphasize that proline should be viewed as a signaling molecule indicating changes in ROS concentration, rather than a direct measure of stress.

## 4. Materials and Methods

### 4.1. Acquisition of Plant Material

Young leaves and shoots of cultivars (cv.) ‘Oblica’ and ‘Pačica’ were collected from a field collection (43.504678, 16.499206), Institute for Adriatic Crops and Karst Reclamation (Split, Croatia). Additional samples of leaves and shoots were also collected from the various locations of olive forests, including the island of Mljet (42.7461361,17.3709178), as we assumed they were wild olive genotypes (*Olea europaea* subsp. *europaea* var. *sylvestris*), based on morphological characteristics [49,50].

### 4.2. Plant Cultivation

After a successful rooting process, three unknown olive genotypes from Mljet island were carefully selected for morphological and biochemical characterization. A period of one year was taken to grow plants in a greenhouse. The olive trees were trained to a single shoot and irrigated daily with a half-strength Hoagland’s nutrient solution [51]. For the experiment, we included cv. ‘Oblica’ as the leading Croatian cultivar in orchards and cv. ‘Pačica’ as less known autochthonous olive from Mljet island. During the three-month adaptation period, plant nutrition was maintained using half-strength Hoagland’s nutrient solution. The nutritional status of daily percolate was monitored via pH measurement using a Mettler Toledo MP 230 pH-meter and electrical conductivity assessment with a Mettler Toledo MC 226 EC-meter (Mettler Toledo, Columbus, OH, USA). To prepare plants for the acclimatization period, they were placed into 3.6 L pots containing a substrate mixture of Agrilite 3 perlite (Perlite Italiana SRL., Milano, Italy) and vermiculite (RHP, Gravenzande, The Netherlands) in a 1:1 (*v*:*v*) ratio, with expanded clay at the bottom to prevent inorganic substrate leakage (Laterlite S.P.A, Milano, Italy). Acclimatization took place in the greenhouse from March to June (3 months) under a natural photoperiod. During this period, the daily minimum and maximum temperatures inside the greenhouse were 16.1 °C and 32.2 °C, respectively.

### 4.3. Experimental Design and Treatment Application

The experimental design was arranged as randomized block experiment, with three iterations. To minimize edge effects and ensure uniform stress application, additional olive plants were placed at the ends of the rows. Controlled environmental conditions were maintained to achieve equivalent values of osmotic potential for 150 mM NaCl and 300 mM mannitol. Those concentrations were selected based on findings from previous research [17,31,34,35]. At the start of the experiment, the plants were exposed to ½ HNS supplemented with 150 mM NaCl (noniodized salt; Solana Pag d.d., Pag, Croatia) and 300 mM mannitol (powder; Roquette, Lestrem, France). Control treatment for each olive genotype involved the application of only ½ HNS following an adaptation process. The treatment lasted for 21 days, with NaCl and mannitol concentrations gradually increased over the first three days to minimize initial osmotic shock. Monitoring the nutritional status of the percolate ensured an accurate assessment of the physiological responses of the plants to the imposed abiotic stresses.

### 4.4. Morphometric Measurements and Ionic Composition Analysis

Morphometric assessments were conducted at three intervals: 1st, 12th and 21st day of the experiment. Shoot measurements were taken from the main vegetative bud of the current year’s growth, which began in March. To maintain consistency, other vegetative shoots from the lateral buds were removed at the start of the vegetative growth stage. Analyses included the assessment of shoot length (cm), leaf surface area (cm^2^), and dry weight (g) of shoots. Epson Perfection V700 Photo scanner and WinFOLIA software v. 2024a were utilized for the shoot and leaf measurements. To obtain dry mass of the plant material, drying process was carried out at 75 °C for 48 h. Ion chromatography (Dionex DX500, Dionex Corporation, Sunnyvale, CA, USA) was used for determination of Na^+^, Cl^−^ and K^+^, while atomic absorption spectrophotometry (SpectraAA 220 AAS, Varian Inc., Palo Alto, CA, USA) was utilized for determination of Ca^2+^ and Mg^2+^ in olive leaves and roots. Ion contents of leaves and roots are expressed in mg/L. Further details are given in Tadić et al. [21].

### 4.5. Biochemical Parameter Analysis

On the final day of the experiment, when the plants’ shoots did not recover from turgor drop, we sampled the first four fully developed leaves from each shoot. It was imperative to capture a representative snapshot of the olives’s biochemical status in peak stress conditions. Before lyophilization (Labconco FreeZone 2.5, Labconco Corporation, Kansas City, MO, USA), liquid nitrogen was used to preserve the biochemical integrity of the sampled leaves. The lyophilization process was carried out at −48 °C and 0.180 mBar until a constant weight was achieved, indicating the removal of all moisture from the samples. Once dried, the samples were stored at a low temperature of −65 °C. Before analysis, the dried samples were homogenized to a fine powder using a mixer mill.

The activities of the antioxidative enzymes were determined by one-minute homogenization of leaf material in 50 mM KPO_4_ buffer (pH 7) 7) including 1 mM ethylene diamine tetraacetic acid (Sigma-Aldrich, Burlington, MA) and polyvinylpolypyrrolidone (Sigma-Aldrich, Burlington, MA). After centrifugation (Sigma 3K18 centrifuge; Osterode am Harz, Germany) at 25,000× *g* for 30 min at 4 °C, further analysis was performed with obtained supernatants. The protein content (mg/g DW) was estimated according to Bradford [52] using bovine serum albumin (Sigma-Aldrich) as standard, and calculated as mg per g of dry weight (mg/g DW). The activity of SOD was assayed by measuring its ability to inhibit the photochemical reduction of nitroblue tetrazolium (Sigma-Aldrich) following the method of Beauchamp and Fridovich [53]. GPOX activity was measured with guaiacol as a substrate [54]. The formation of tetraguaiacol was followed at 430 nm and was quantified taking its extinction coefficient (26.6 mM/cm) into account. The specific enzyme activity for enzymes was expressed as units per milligram of protein (U/mg protein). Lipid peroxidation was evaluated by estimation of malondialdehyde (MDA). The reagent solution contains thiobarbituric acid (TBA) that reacts with MDA forming the MDA-TBA adduct. The absorbance of the complex was measured at 532 nm and the results are given as nmol per mg DW (nmol/mg DW) [55]. Free proline content was measured using the ninhydrin solution following the absorbance at 520 nm and the results are expressed as μmol per g DW (μmol/g DW) [55]. Photosynthetic pigments were estimated in acetone (80%) extracts [56] and the the results are given as mg per g of dry weight (mg/g DW).

### 4.6. Statistical Analysis

Statistical analysis was carried out using STATISTICA 13.3 (TIBCO, Inc.,Palo Alto, CA) and Microsoft Office Excel. Each data point is the average of three biological replicates ± standard deviation (SD). To ensure fair comparison among olive genotypes, all data were normalized to controls (set to 1) of respective olive genotypes. One-way analysis of variance (ANOVA) was utilized to determine statistical significance among the treated samples, with post hoc Duncan’s multiple range test (*p* ≤ 0.05). PCA was performed for all measured variables in all tested genotypes (M27, M28, M29, Oblica, Pačica) using R version 4.0.2 and the ‘factoextra’ package. To reduce data noise, values were normalized to control (set to 1), with only one control group included in the analysis. The biplot was constructed using the first two principal components. The original dataset can be found in Supplementary Materials.

## 5. Conclusions

Olive trees (*Olea europaea* L.) face significant challenges from abiotic stress factors such as drought and salinity, which affect their morphological, physiological and biochemical traits. Multivariate analysis of drought- and salinity-treated plants showed that the treatment conditions—drought and salinity—were the most important factors influencing the data variations, overshadowing the genetic differences between the selected olive genotypes. In particular, drought had a stronger effect compared to salinity, leading to greater reductions in growth parameters and chlorophyll *a* content, indicating reduced photosynthetic efficiency and overall plant health. However, cv. ‘Oblica’ and genotype M29 proved to be more resistant to salinity and managed to maintain shoot length and leaf surface area better than other genotypes. The ability of more tolerant genotypes to cope with salinity is attributed to their efficient ion storage and distribution mechanisms. For example, cv. ‘Oblica’ demonstrated superior resilience by translocating Na^+^ ions to vacuoles, thereby protecting sensitive tissues from damage. In addition, enzyme activities, such as those of SOD and GPOX, play a key role in the antioxidant defense system during adverse environmental conditions. Cv. ‘Oblica’ had higher SOD activity compared to other genotypes, which probably contributes to its resistance to salinity.

The research suggests the resilience displayed by cultivated olive types such as cv. ‘Oblica’ and M29 differ from that of more susceptible genotypes, including wild olives (*Olea europaea* subsp. *europaea* var. *sylvestris*), as outlined in the prior research conducted by Tadić et al. [22]. Thus, it can be concluded that resistance to abiotic stress is a genotype- dependent trait regardless of subspecies variety origin. The resilience discovered in genotypes from cultivated origins on the Mljet island, with advancements in genetic enhancements, paves the way for improved stress resistance in olives and supports sustainable cultivation practices. A thorough understanding of how different olive genotypes respond to stress—assessed through morphometric, physiological, and biochemical parameters—shows the importance of selecting stress-resistant cultivars for breeding more resilient genotypes. These insights are key to developing robust olive cultivars that can thrive in challenging environments. In summary, the overall findings underscore the need for well-researched strategies to address olive cultivation in response to changing climate conditions. The use of genetic diversity, biotechnological advances, and targeted breeding will be key to developing stress-resilient olive cultivars, thus ensuring productivity and sustainability in the face of environmental challenges.

## Figures and Tables

**Table 1:** Relative ion content (Mg^2+^, Ca^2+^, K^+^) and K^+^ leakage in shoot leaves of olive trees under short-term salinity and drought.

Genotype	Treatment	Mg^2+^ (mg/L)	Ca^2+^ (mg/L)	K^+^ (mg/L)	K^+^ Leakage (mg/L)
Oblica	control *	1.00 ± 0.038 ^a^	1.00 ± 0.063 ^a^	1.00 ± 0.189 ^a^	1.00 ± 0.171 ^bc^
	salinity	0.73 ± 0.081 ^bc^	0.36 ± 0.016 ^b^	0.87 ± 0.038 ^bc^	2.90 ± 0.674 ^a^
	drought	0.55 0.009 ^d^	0.27 ± 0.011 ^b^	1.01 ± 0.059 ^a^	1.72 ± 0.020 ^b^
	control	1.00 ± 0.191 ^a^	1.00 ± 0.059 ^a^	1.00 ± 0.045 ^a^	1.00 ± 0.172 ^bc^
M27	salinity	0.80 ± 0.016 ^ab^	1.11 ± 0.397 ^a^	0.98 ± 0.026 ^a^	1.07 ± 0.393 ^bc^
	drought	0.94 ± 0.025 ^a^	1.20 ± 0.167 ^a^	0.88 ± 0.023 ^abc^	0.97 ± 0.225 ^bc^
	control	1.00 ±0.448 ^a^	1.00 ± 0.119 ^a^	1.00 ± 0.024 ^a^	1.00 ± 0.218 ^bc^
M28	salinity	0.70 ±0.035 ^cd^	0.82 ± 0.198 ^ab^	0.95 ± 0.019 ^ab^	1.60 ± 0.613 ^b^
	drought	0.97 ± 0.214 ^a^	1.14 ± 0.198 ^a^	1.01 ± 0.213 ^a^	0.60 ± 0.302 ^c^
	control	1.00 ± 0.291 ^a^	1.00 ± 0.100 ^a^	1.00 ± 0.047 ^a^	1.00 ± 0.155 ^bc^
M29	salinity	0.73 ± 0.027 ^bc^	0.83 ± 0.164 ^ab^	0.90 ± 0.012 ^ab^	1.83 ± 0.648 ^b^
	drought	0.79 ± 0.030 ^ab^	1.09 ± 0.092 ^a^	0.88 ± 0.017 ^abc^	1.39 ± 0.288 ^bc^
	Control	1.00 ± 0.397 ^a^	1.00 ± 0.150 ^a^	1.00 ± 0.172 ^a^	1.00 ± 0. 581 ^bc^
Pačica	Salinity	0.93 ± 0.041 ^a^	0.85 ± 0.071 ^ab^	0.93 ± 0.022 ^ab^	2.08 ± 0.652 ^ab^
	Drought	0.94 ± 0.020 ^a^	1.31 ± 0.338 ^a^	0.79 ± 0.006 ^c^	1.60 ± 0.443 ^b^

* Controls of olive genotypes are set to the value 1. Presented data are means of three replicates ± SD. Different letters within the columns are significantly different at *p* ≤ 0.05. Raw data (absolute values) are presented in Supplementary Table S4.

**Table 2:** Relative ion content (Mg^2+^, Ca^2+^, K^+^) in roots of olive trees under short-term salinity and drought.

Genotype	Treatment	Mg^2+^ (mg/L)	Ca^2+^ (mg/L)	K^+^ (mg/L)
Oblica	control *	1.00 ± 0.113 ^cd^	1.00 ± 0.168 ^d^	1.00 ± 0.011 ^a^
	salinity	0.95 ± 0.162 ^cd^	0.89 ± 0.223 ^d^	0.90 ± 0.041 ^b^
	drought	0.93 ± 0.009 ^cd^	1.28 ± 0.089 ^c^	0.66 ± 0.039 ^e^
	control	1.00 ± 0.062 ^cd^	1.00 ± 0.048 ^d^	1.00 ± 0.0330 ^a^
M27	salinity	1.29 ± 0.055 ^ab^	0.56 ± 0.042 ^e^	0.71 ± 0.011 ^cde^
	drought	1.40 ± 0.127 ^a^	1.01 ± 0.082 ^cd^	0.76 ± 0.062 ^c^
	control	1.00 ± 0.048 ^cd^	1.00 ± 0.049 ^d^	1.00 ± 0.034 ^a^
M28	salinity	0.95 ± 0.121 ^cd^	1.11 ± 0.054 ^cd^	0.69 ± 0.011 ^de^
	drought	1.10 ± 0.066 ^bc^	2.25 ± 0.182 ^a^	0.59 ± 0.056 ^f^
	control	1.00 ± 0.049 ^cd^	1.00 ± 0.036 ^d^	1.00 ± 0.036 ^a^
M29	salinity	0.86 ± 0.045 ^cd^	0.83 ± 0.024 ^d^	0.76 ± 0.032 ^c^
	drought	0.78 ± 0.069 ^d^	1.05 ± 0.077 ^cd^	0.51 ± 0.015 ^g^
	control	1.00 ± 0.022 ^cd^	1.00 ± 0.072 ^d^	1.00 ± 0.024 ^a^
Pačica	salinity	0.93 ± 0.129 ^cd^	0.38 ± 0.032 ^e^	0.73 ± 0.017 ^cd^
	drought	1.43 ± 0.247 ^a^	1.65 ± 0.362 ^b^	0.55 ± 0.016 ^fg^

* Controls of olive genotypes are set to the value 1. Presented data are means of three replicates ± SD. Different letters within the columns are significantly different values at *p* ≤ 0.05. Raw data (absolute values) are presented in Supplementary Table S5.

## Data Availability

The original data presented in the study are available in Supplementary Materials.

## Supplementary Materials

The following supporting information can be downloaded at: https://www.mdpi.com/article/10.3390/plants13182549/s1, Figure S1: Principal component analysis (PCA) biplot of olive response to drought and salinity. Biplot illustrates the relationship between different olive genotypes (M27, M28, M29, Oblica, Pačica) and the measured morphological (shoot length and dry mass, leaf surface area), physiological (chlorophyll *a*, chlorophyll *b* and total carotenoids content) and biochemical (SOD and GPOX activity, proline and MDA content, K^+^ leakage, Na^+^, Cl^−^, K^+^, Mg2^+^ and Ca2^+^ ion content in leaves and roots) variables under drought and salinity. Controls are normalized to the value 1. Each genotype is represented by three biological replicates. Abbreviations: D1–3—biological replicates exposed to drought, O—Oblica, PA—Pačica, S1–3—biological replicates exposed to salinity.; Table S1: Morphometric parameters; Table S2: Photosynthetic parameters; Table S3: Sodium and chlorine content in olive leaves and roots; Table S4: Mineral ion content in olive leaves; Table S5: Mineral ion content in olive roots; Table S6: Biochemical parameters in olive leaves.
