# Supplementary material for: Challenges of Salinity Intrusion and Drought Stress on Olive Tree Cultivation on Mljet Island
Source: Plants (Basel). 2024 Sep 11;13(18):2549. doi: 10.3390/plants13182549 (PMC11435225; doi:10.3390/plants13182549)
Supplement: Supplementary file 1 [file plants-13-02549-s001.zip › plants-3165024-supplementary.pdf]

Supplementary Material

Challenges of Salinity Intrusion and Drought Stress on Olive Tree Cultivation on Mljet island

Josip Tadić [1,2], Gvozden Dumičić [1], Maja Veršić Bratinčević [1], Sandra Vitko [3] and Sandra Radić Brkanac [3,*]

[1] Institute for Adriatic Crops and Karst Reclamation, Split, Croatia; josip.tadic@krs.hr; gvozden.dumicic@krs.hr; Maja.Versic.Bratincevic@krs.hr;

[2] Centre of Excellence for Biodiversity and Molecular Plant Breeding (CoE CroPBioDiv), Zagreb, Croatia; josip.tadic@krs.hr

[3] Faculty of Science, University of Zagreb, Zagreb, Croatia; sandra.vitko@biol.pmf.hr; sandra.radic.brkanac@biol.pmf.hr;

* Correspondence: sandra.radic.brkanac@biol.pmf.hr;

Supplementary Table 1: Morphometric parameters

Genotype	Treatment	Shoot length (cm)	Leaf surface area (cm^2)	Shoot dry mass (g)
**Oblica**	Control	18.63 (10.17)	59.08 (34.62)	0.42 (0.40)
	Salinity	20.83 (3.56)	58.81 (12.08)	0.35 (0.09)
	Drought	10.03 (1.21)	20.52 (7.40)	0.13 (0.03)
**M27**	Control	33.37 (7.81)	43.99 (15.31)	0.67 (0.21)
	Salinity	16.17 (5.65)	20.25 (3.27)	0.25 (0.11)
	Drought	15.27 (2.03)	23.40 (7.04)	0.22 (0.04)
**M28**	Control	24.63 (3.37)	78.88 (38.99)	0.43 (0.14)
	Salinity	18.77 (4.26)	27.18 (13.52)	0.25 (0.05)
	Drought	10.10 (3.29)	22.39 (11.19)	0.14 (0.07)
**M29**	Control	15.27 (5.26)	28.74 (12.76)	0.22 (0.06)
	Salinity	16.33 (2.92	32.05 (17.32)	0.19 (0.04)
	Drought	11.83 (1.96)	17.68 (5.19)	0.12 (0.03)
**Pačica**	Control	25.47 (0.86)	82.65 (19.87)	0.75 (0.13)
	Salinity	14.63 (5.35)	44.37 (40.92)	0.31 (0.07)
	Drought	12.80 (3.14)	23.62 (5.51)	0.24 (0.11)

Data are presented as averages (SD in parenthesis), n=3

Supplementary Table 2: Photosynthetic parameters

Genotype	Treatment	Chlorophyll *a* (mg/g DW)	Chlorophyll *b* (mg/g DW)	Carotenoids (mg/g DW)
**Oblica**	Control	3.12 (0.40)	0.77 (0.08)	1.20 (0.15)
	Salinity	2.81 (0.46)	0.62 (0.09)	1.11 (0.14)
	Drought	1.91 (0.28)	0.71 (0.13)	1.03 (0.11)
**M27**	Control	4.29 (0.57)	2.35 (0.49)	1.61 (0.19)
	Salinity	2.22 (0.13)	1.29 (0.24)	0.88 (0.05)
	Drought	2.78 (0.59)	1.81 (0.63)	1.22 (0.13)
**M28**	Control	3.27 (0.12)	1.85 (0.09)	1.25 (0.02)
	Salinity	3.12 (0.18)	2.59 (0.72)	1.23 (0.06)
	Drought	2.80 (0.40)	1.95 (0.49)	1.18 (0.15)
**M29**	Control	3.70 (0.26)	2.65 (0.35)	1.45 (0.07)
	Salinity	3.78 (0.32)	2.48 (0.33)	1.47 (0.12)
	Drought	3.16 (0.64)	2.45 (0.29)	1.35 (0.23)
**Pačica**	Control	4.05 (0.74)	2.41 (0.43)	1.50 (0.24)
	Salinity	4.40 (0.83)	2.55 (0.66)	1.63 (0.29)
	Drought	2.86 (0.22)	1.04 (0.07)	1.28 (0.20)

Data are presented as averages (SD in parenthesis), n=3

Supplementary Table 3: Sodium and Chlorine Content in Olive Leaves and Roots

Genotype	Treatment	Leaf Na^+ (mg/L)	Root Na^+ (mg/L)	Leaf Cl^- (mg/L)	Root Cl^- (mg/L)
**Oblica**	Control	0.31 (0.08)	6.19 (1.88)	9.62 (1.86)	6.19 (1.88)
	Salinity	21.95 (0.38)	68.20 (12.22)	28.08 (1.38)	68.20 (12.22)
	Drought	0.38 (0.04)	2.92 (0.07)	6.78 (0.23)	2.92 (0.07)
**M27**	Control	0.34 (0.06)	4.70 (0.44)	4.47 (0.21)	12.01 (3.86)
	Salinity	3.04 (0.20)	114.61 (3.58)	7.94 (0.40)	97.05 (3.65)
	Drought	0.87 (0.67)	9.06 (7.10)	4.48 (0.50)	5.38 (1.12)
**M28**	Control	0.35 (0.19)	5.60 (0.17)	3.65 (0.19)	16.36 (1.04)
	Salinity	10.40 (1.31)	98.55 (4.27)	12.52 (0.72)	158.55 (4.49)
	Drought	0.62 (0.31)	6.35 (0.82)	3.11 (0.37)	8.10 (0.30)
**M29**	Control	0.30 (0.09)	6.64 (1.61)	4.04 (0.55)	12.47 (4.37)
	Salinity	0.84 (0.15)	123.76 (8.93)	6.17 (0.16)	78.85 (28.39)
	Drought	0.43 (0.08)	7.75 (4.76)	5.44 (0.21)	5.41 (2.87)
**Pačica**	Control	0.53 (0.21)	5.45 (1.61)	4.30 (1.69)	10.97 (4.37)
	Salinity	1.72 (1.11)	123.76 (8.93)	10.93 (0.91)	150.91 (16.67)
	Drought	0.37 (0.03)	4.97 (0.65)	4.48 (0.51)	4.07 (0.90)

Data are presented as averages (SD in parenthesis), n=3

Supplementary Table 4: Mineral Ion Content in Olive Leaves

Genotype	Treatment	Mg^+ (mg/L)	Ca^+ (mg/L)	K^+ (mg/L)	K^+ leaching (mg/L)
**Oblica**	Control	1.60 (0.05)	4.20 (0.07)	67.44 (3.27)	5.01 (0.85)
	Salinity	1.16 (0.13)	3.51 (0.19)	58.66 (2.55)	14.54 (3.37)
	Drought	0.88 (0.01)	2.62 (0.11)	68.20 (3.95)	8.63 (0.10)
**M27**	Control	3.59 (0.52)	3.47 (0.21)	66.46 (3.02)	5.33 (0.92)
	Salinity	2.88 (0.06)	3.86 (1.38)	64.98 (1.72)	5.69 (2.10)
	Drought	3.37 (0.09)	4.16 (0.58)	58.40 (1.50)	5.15 (1.20)
**M28**	Control	5.69 (0.81)	5.62 (0.67)	119.15 (2.79)	8.52 (1.86)
	Salinity	4.00 (0.20)	4.63 (1.12)	113.12 (2.28)	13.67 (3.34)
	Drought	5.51 (2.36)	6.42 (4.65)	120.01 (25.41)	5.15 (2.58)
**M29**	Control	4.18 (0.24)	3.86 (0.39)	59.56 (2.78)	5.89 (1.69)
	Salinity	3.05 (0.11)	3.19 (0.63)	53.84 (0.70)	10.76 (4.41)
	Drought	3.31 (0.13)	4.19 (0.35)	52.47 (1.02)	8.18 (1.37)
**Pačica**	Control	5.84 (1.12)	6.58 (0.99)	145.33 (25.14)	5.03 (2.23)
	Salinity	5.44 (0.24)	5.59 (0.47)	134.89 (3.22)	10.48 (4.79)
	Drought	5.49 (0.12)	8.61 (3.55)	114.54 (0.86)	8.05 (2.17)

Data are presented as averages (SD in parenthesis), n=3

Supplementary Table 5: Mineral Ion Content in Olive Roots

Genotype	Treatment	Mg^+ (mg/L)	Ca^+ (mg/L)	K^+ (mg/L)
**Oblica**	Control	2.81 (0.32)	11.67 (1.97)	49.26 (0.54)
	Salinity	2.66 (0.46)	10.35 (2.60)	44.47 (2.00)
	Drought	2.61 (0.03)	14.90 (1.04)	32.57 (1.91)
**M27**	Control	7.25 (0.45)	48.58 (2.33)	90.19 (2.99)
	Salinity	9.36 (0.40)	27.16 (2.05)	64.03 (0.96)
	Drought	10.15 (0.92)	49.07 (3.98)	68.12 (5.55)
**M28**	Control	8.25 (0.39)	8.25 (0.41)	94.99 (3.28)
	Salinity	7.86 (1.00)	9.13 (0.45)	65.36 (1.04)
	Drought	9.05 (0.54)	18.55 (1.50)	56.20 (1.47)
**M29**	Control	12.39 (0.61)	35.41 (1.29)	105.10 (3.81)
	Salinity	10.97 (0.56)	29.53 (0.85)	79.94 (3.35)
	Drought	9.63 (0.85)	37.05 (2.74)	53.98 (1.60)
**Pačica**	Control	7.94 (0.17)	30.11 (2.16)	58.68 (1.43)
	Salinity	7.38 (1.03)	11.36 (0.95)	42.56 (0.99)
	Drought	11.34 (1.96)	49.71(10.89)	32.21 (0.93)

Data are presented as averages (SD in parenthesis), n=3

Supplementary Table 6: Biochemical parameters in olive leaves

Genotype	Treatment	SOD U/mg protein	GPOD U/mg protein	MDA (nmol/g DW)	Proline (μmol/g DW)
**Oblica**	Control	120.30 (4.32)	1.09 (0.10)	159.87 (13.84)	0.18 (0.01)
	Salinity	172.25 (12.00)	0.34 (0.08)	157.07 (11.55)	0.14 (0.03)
	Drought	173.25 (4.00)	0.44 (0.02)	149.20 (16.41)	0.19 (0.07)
**M27**	Control	243.59 (37.75)	1.23 (0.10)	121.28 (9.86)	0.22 (0.03)
	Salinity	268.68 (27.52)	0.76 (0.07)	101.08 (5.32)	0.32 (0.04)
	Drought	204.86 (16.88)	1.73 (0.11)	183.16 (29.14)	0.33 (0.06)
**M28**	Control	311.78 (17.55)	0.15 (0.01)	98.62 (16.46)	0.34 (0.02)
	Salinity	222.48 (14.39)	0.17 (0.00)	93.69 (8.46)	0.32 (0.04)
	Drought	313.38 (4.10)	0.24 (0.03)	98.31 (1.85)	0.36 (0.02)
**M29**	Control	277.60 (32.42)	0.34 (0.03)	101.21 (17.00)	0.33 (0.02)
	Salinity	285.68 (8.49)	1.42 (0.24)	124.61 (9.49)	0.53 (0.01)
	Drought	161.77 (20.94)	0.67 (0.08)	139.87 (18.02)	0.47 (0.05)
**Pačica**	Control	361.52 (28.99)	3.27 (0.11)	88.56 (6.34)	0.27 (0.02)
	Salinity	260.80 (21.07)	2.79 (0.09)	111.83 (20.37)	0.38 (0.03)
	Drought	601.30 (26.25)	2.04 (0.20)	109.17 (6.23)	0.41 (0.04)

Data are presented as averages (SD in parenthesis), n=3

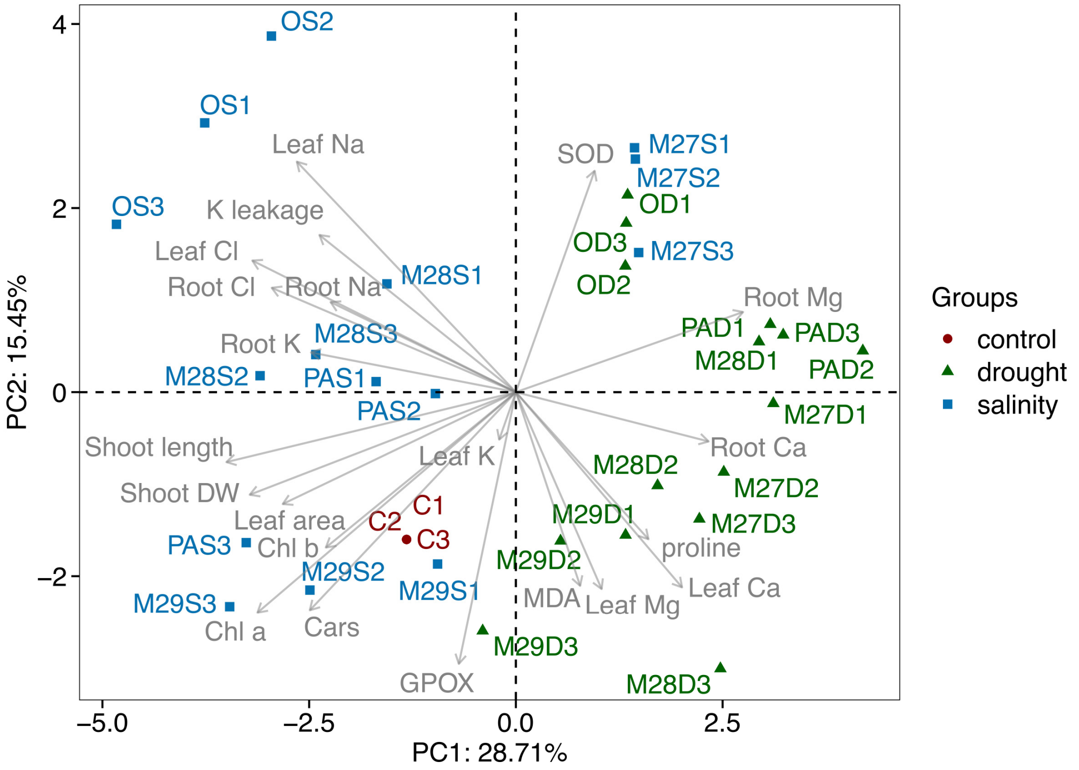

Figure S1. Principal component analysis (PCA) biplot of olive response to drought and salinity. Biplot illustrates the relationship between different olive genotypes (M27, M28, M29, Oblica, Pačica) and the measured morphological (shoot length and dry mass, leaf surface area), physiological (chlorophyll *a*, chlorophyll *b* and total carotenoids content) and biochemical (SOD and GPOX activity, proline and MDA content, K^+ leakage, Na^+, Cl^-, K^+, Mg^{2+} and Ca^{2+} ion content in leaves and roots) variables under drought and salinity. Controls are normalized to the value 1. Each genotype is represented by three biological replicates. Abbreviations: D1-3 – biological replicates exposed to drought, O – Oblica, PA – Pačica, S1-3 – biological replicates exposed to salinity.
